# Causal Associations of Heparin‐Binding Growth and Differentiation Factors With Thyroid Cancer: A Two‐Sample Mendelian Randomization Study

**DOI:** 10.1155/ije/6165618

**Published:** 2026-07-15

**Authors:** Xiwei Zhang, Dongqiang Yang, Yan Liu, Yanzhao Wu, Ping Shi

**Affiliations:** ^1^ Department of Head and Neck Surgical Oncology, National Cancer Center/National Clinical Research Center for Cancer/Cancer Hospital, Chinese Academy of Medical Sciences and Peking Union Medical College, Beijing, 100034, China, cacms.ac.cn; ^2^ Department of Radiological Intervention, The Fourth Hospital of Hebei Medical University and Hebei Tumor Hospital, Shijiazhuang, 050000, China; ^3^ Department of Otolaryngology Head and Neck Surgery, The Fourth Hospital of Hebei Medical University and Hebei Tumor Hospital, Shijiazhuang, 050000, China

**Keywords:** heparin-binding EGF-like growth factor, heparin-binding growth/differentiation factors, IGF2, Mendelian randomization, midkine, thyroid cancer

## Abstract

**Background:**

Heparin‐binding growth and differentiation factors (GDFs) play roles in various cellular processes and are potential contributors to thyroid cancer. Although population‐based studies have documented associations between heparin‐binding GDFs and thyroid cancer, their causal relationships remain unclear.

**Methods:**

A two‐sample Mendelian randomization (MR) analysis was conducted using published genome‐wide association studies (GWASs) data. The primary method for estimating causal effects was the inverse‐variance weighted (IVW) approach, supplemented by multiple sensitivity analyses including weighted median, MR‐Egger, and MR‐PRESSO. Heterogeneity and outlier effects were systematically evaluated. Additionally, key MR findings were validated at the transcriptomic level using differential expression analysis of data from the Cancer Genome Atlas (TCGA).

**Results:**

The IVW method revealed significant causal relationships between midkine levels (OR = 1.2099, 95% CI: 1.013–1.445, *p* = 0.0355) and IGF2 (OR = 0.7496, 95% CI: 0.6056–0.9279, *p* = 0.0081) with thyroid cancer and between heparin‐binding EGF‐like growth factor and malignant thyroid neoplasms (OR = 0.8821, 95% CI: 0.7873–0.9882, *p* = 0.0304). Heterogeneity was identified in the association between FGF1 and malignant thyroid neoplasms (*Q* = 20.725, *p* = 0.036). Neither MR‐Egger analysis nor the MR‐PRESSO global test found evidence of horizontal pleiotropy in the association between heparin‐binding GDFs and thyroid cancer. The robustness of these findings was supported by sensitivity analyses, and transcriptomic analysis of TCGA data further revealed that midkine (*MDK* was significantly upregulated in thyroid tumor tissues.

**Conclusions:**

Genetically predicted midkine levels and IGF2 were associated with thyroid cancer, and heparin‐binding EGF‐like growth factor was associated with malignant thyroid neoplasms. Future studies are needed to validate these findings.

## 1. Introduction

Thyroid cancer, the most common endocrine malignancy, includes various histological subtypes such as papillary, follicular, medullary, and anaplastic thyroid carcinomas (THCAs) [1]. Global prevalence of thyroid cancer was estimated to be 586,000 cases in 2020, which ranked as the 10th most prevalent cancer [2]. Notably, anaplastic THCA, though rare, is highly aggressive, with a median survival of just 3–6 months and a 1‐year survival rate of less than 20% [3, 4]. While differentiated thyroid cancers like papillary and follicular types have relatively high survival rates, the disease often imposes significant physical and psychological burdens due to the need for lifelong monitoring, anxiety over recurrence, and treatments that can reduce quality of life [4, 5]. This underscores the importance of understanding the etiological factors of thyroid cancer, which remains a critical area of ongoing research. These issues emphasize the critical need for a deeper understanding of the etiological factors of thyroid cancer, which continues to be a central area of research.

Recent research has highlighted the role of heparin‐binding growth and differentiation factors (GDFs), a group of proteins involved in cellular processes including proliferation, differentiation, and angiogenesis [6]. Heparin‐binding GDFs can bind to heparin and heparan sulfate proteoglycan and trigger downstream signaling pathways of cellular homeostasis and healing processes [7, 8]. For instance, fibroblast growth factor 1 (FGF1) and FGF2 are critical in wound healing and angiogenesis [9], whereas insulin‐like growth factor I (IGF1) and IGF2 are crucial for growth and development [10]. Midkine and pleiotrophin are known for their roles in neurogenesis and cancer progression [11], while thyroglobulin is a key marker in thyroid function and thyroid cancer monitoring [12]. Population‐based studies have provided evidence supporting the associations between GDFs and thyroid cancer. For instance, serum midkine and thyroglobulin can serve as effective biomarkers for screening thyroid nodules for differentiated thyroid cancer preoperatively [13]. However, the presence of confounding factors and reverse causation in observational studies has made it difficult to establish a clear causal link between heparin‐binding GDFs and thyroid cancer. This challenge highlights the need for more rigorous approaches to better clarify the potential causal relationships.

Mendelian randomization (MR) analysis utilizes genetic variants as proxies for modifiable exposures to infer causal relationships between exposures and outcomes in observational studies [14]. This approach is grounded in the principle that genetic variants are randomly assorted during gamete formation and thus are generally independent of confounding factors in observational studies. MR analysis assumes that the genetic variants used as instruments are associated with the exposure, are not associated with any confounders of the exposure‐outcome relationship, and influence the outcome solely through the exposure [15].

To investigate the potential causal links between heparin‐binding GDFs with thyroid cancer, a two‐sample MR analysis was conducted in this study. Elucidating the biological mechanisms underlying the causal association can enhance our understanding of thyroid cancer pathogenesis, potentially leading to novel prevention strategies and therapeutic interventions.

## 2. Materials and Methods

### 2.1. Study Design

**FIGURE 1:**
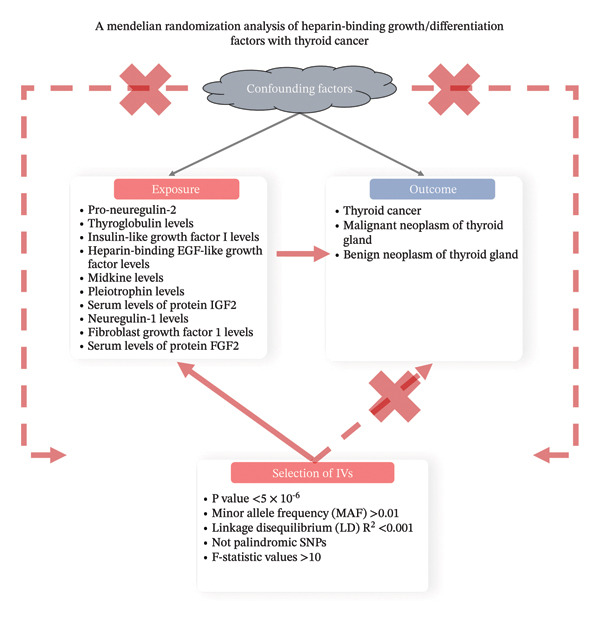
Flowchart of the study design.

A two‐sample MR study was conducted to investigate the causal links between heparin‐binding GDFs and thyroid cancer. Utilizing data from previously published studies, genetic variants related to heparin‐binding GDFs were collected. Instrumental variables (IVs) were selected according to three core assumptions [15]: (A) the single nucleotide polymorphism (SNP) is strongly associated with heparin‐binding GDFs, (B) the SNP is independent of known confounders, and (C) the SNP affects thyroid cancer exclusively through its impact on heparin‐binding GDFs. The study methodology is illustrated in Figure 1.

### 2.2. Data Sources

Summary data for thyroid cancer were obtained from the IEU (https://gwas.mrcieu.ac.uk/datasets/) and FinnGen (https://storage.googleapis.com/finngen-public-data-r10/summary_stats/) database, including subtypes such as malignant and benign neoplasms of the thyroid gland (Table S1). Summary statistics for proneuregulin‐2, thyroglobulin, IGF‐1, heparin‐binding EGF‐like growth factor, midkine, pleiotrophin, IGF2, neuregulin‐1, FGF1, and FGF2 levels were sourced from the genome‐wide association studies (GWASs) catalog (Table S1). To minimize population stratification bias, all SNPs and summary data were derived exclusively from European ancestry populations. The exposure GWASs were conducted in independent European cohorts, while outcome data for malignant thyroid neoplasms came from the FinnGen project (Finnish population) and those for overall thyroid cancer and benign neoplasms from the IEU database (primarily UK Biobank). There is no substantial sample overlap between exposure and outcome datasets. Using open‐source data from ethically compliant sources, such as those adhering to the Helsinki Declaration, does not require additional ethical approval.

### 2.3. Selection of IVs

SNPs strongly associated with heparin‐binding GDFs were screened based on a *p* value threshold of < 5 × 10^−6^ [16]. This threshold, rather than the conventional genome‐wide significance level of *p* < 5 × 10^−8^, was adopted because for several exposures the number of independent SNPs meeting the stricter cutoff was fewer than three. An insufficient number of IVs would preclude essential sensitivity analyses, including MR‐Egger regression and the MR‐PRESSO global test. To mitigate potential weak instrument bias associated with the relaxed threshold, F‐statistics were calculated for all included SNPs, and only those exceeding the empirical benchmark of 10 were retained. SNPs with a minor allele frequency (MAF) ≤ 0.01 were additionally excluded [17]. Linkage disequilibrium (LD) analysis removed SNPs with *R* ^2^ < 0.001 (window size = 10,000 kb) [18]. For outcome summary data, missing SNPs were replaced with proxy SNPs showing high LD (*R* ^2^ > 0.8). Proxy SNPs were identified using the LDlink web–based tool (https://ldlink.nih.gov/?tab=ldproxy) and were selected based on an *R* ^2^ > 0.8 threshold in the European 1000 Genomes Project population. This high degree of LD ensures that the proxy SNP serves as a reliable surrogate for the original variant, introducing minimal bias to the causal estimates. If no suitable proxy meeting this criterion was identified, the SNP was excluded from the analysis. Palindromic SNPs were eliminated to avoid discrepancies in strand orientation or allele coding. The F‐statistic is calculated using the following formula:

(1) F=R2×N−21−R2,

where *N* denotes the sample size of the GWAS, and *R* ^2^ represents the proportion of variance in the exposure explained by the IVs in the regression equation [19].

### 2.4. MR Analysis

The primary method for assessing causal relationships between heparin‐binding GDFs and thyroid cancer was the inverse‐variance weighted (IVW) approach. IVW calculates a weighted average effect size, with the inverse variance of each SNP serving as the weight [20]. To validate the findings, additional analyses were conducted using MR‐Egger, weighted median, and weighted mode methods. MR‐Egger adjusts for pleiotropy by accounting for intercept terms, providing precise causal effect estimates [20]. The weighted median method assumes that at least half of the IVs are valid [21], while the weighted mode method determines the mode of effect estimates from individual IVs and assigns appropriate weights [22]. All analyses were performed using the “TwoSampleMR” package in R software (Version 4.0.5).

### 2.5. Sensitivity Analysis

Cochran’s Q test was used to assess heterogeneity based on IVW estimates, with a significance threshold of *p* < 0.05 [23]. A “leave‐one‐out” sensitivity analysis was conducted to identify potentially influential SNPs by iteratively excluding each SNP [24]. MR‐Egger regression was used to evaluate potential horizontal pleiotropy from genetic variation, with a near‐zero or statistically insignificant intercept term indicating the absence of pleiotropy [25]. The MR pleiotropy residual sum and outlier (MR‐PRESSO) method identified outliers (i.e., SNPs with *p* < 0.05), and their removal allowed for re‐estimation of causal associations, thus correcting for horizontal pleiotropy [26]. Asymmetry in the funnel plot can indicate heterogeneity among IVs.

### 2.6. Transcriptomic Validation Using the Cancer Genome Atlas (TCGA) Data

To validate our MR findings at the tissue expression level, we performed a differential expression analysis using data from the TCGA–THCA project. RNA‐sequencing data and corresponding clinical information for 519 thyroid cancer patients and 59 normal control samples were downloaded from the UCSC Xena database (https://xenabrowser.net/datapages/). The limma package (v3.62.2) in R (v4.4.2) was used to identify differentially expressed genes between tumor and normal tissues. *p* values were adjusted for multiple testing using the Benjamini–Hochberg method, with an adjusted *p* value (p.adj) < 0.05 considered statistically significant.

## 3. Results

### 3.1. Selection of IVs

For heparin‐binding GDFs, 163 relevant IVs were identified, with F‐statistics ranging from 20.93 to 79.47 (mean = 24.2). Two IVs were excluded due to the absence of proxy SNPs in the genetic summary for thyroid cancer. Additionally, 9 IVs were missing from the genetic summaries for malignant and benign neoplasms of the thyroid gland. Among these, rs60229479 was substituted by proxy SNP rs6079365, and rs35660775 was replaced with rs10413312. Detailed information on the IVs used is provided in Table S2.

### 3.2. Causal Effects of Heparin‐Binding GDFs on Thyroid Cancer

The IVW method revealed significant causal relationships between midkine levels (OR = 1.2099, 95% CI: 1.013–1.445, *p* = 0.0355) and IGF2 (OR = 0.7496, 95% CI: 0.6056–0.9279, *p* = 0.0081) with thyroid cancer and between heparin‐binding EGF‐like growth factor and malignant thyroid neoplasms (OR = 0.8821, 95% CI: 0.7873–0.9882, *p* = 0.0304) (Table 1). Although the alternative MR methods (including MR‐Egger, weighted median, and weighted mode) did not yield statistically significant *p* values, they demonstrated consistent directional trends with the primary IVW estimates, with all odds ratios (ORs) pointing in the same direction (Table S3). Specifically, for midkine levels, the risk estimates were consistently greater than 1.0 across all methods, while for IGF2 and heparin‐binding EGF‐like growth factor, the estimates remained consistently below 1.0. Scatter plots illustrating SNP effect sizes for heparin‐binding GDFs on thyroid cancer, malignant thyroid neoplasms, and benign thyroid neoplasms are shown in Figures 2. Consistent results are presented in the forest plots (Figures 3).

**TABLE 1:** MR estimates of assessing the causal effects of heparin‐binding GDFs on thyroid cancer by inverse‐variance weighted method.

Outcome	Exposure	Number of SNPs	OR (95% CI)	*p*
Thyroid cancer	Midkine levels	14	1.2099 (1.013–1.445)	0.0355
Thyroid cancer	Serum levels of protein IGF2	21	0.7496 (0.6056–0.9279)	0.0081
Malignant neoplasm of thyroid gland	Heparin‐binding EGF‐like growth factor levels	20	0.8821 (0.7873–0.9882)	0.0304

FIGURE 2The causal relationships of heparin‐binding GDFs with thyroid cancer using different MR methods. Each panel represents the causal estimates for (A) midkine levels and (B) serum levels of protein IGF2 on thyroid cancer and (C) heparin‐binding EGF‐like growth factor levels on malignant neoplasm of thyroid gland. The slope of each line corresponds to the causal estimates for each method. Individual SNP effects on the outcome (represented by points and vertical lines) against their effects on the exposure (represented by points and horizontal lines) are delineated in the background.

**Figure:**
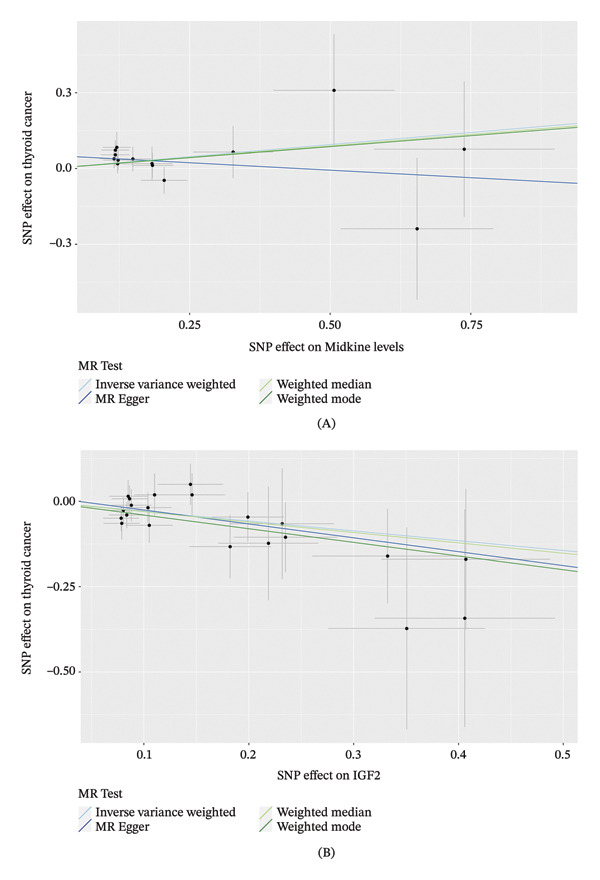

**Figure:**
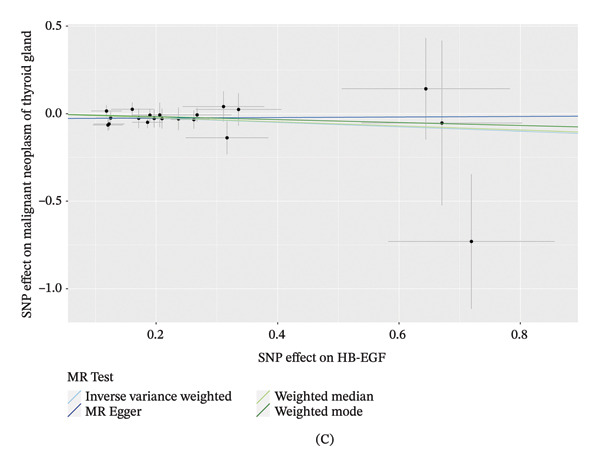

FIGURE 3Forrest plot of the causal relationships of heparin‐binding GDFs with thyroid cancer. Each panel represents the causal estimates for (A) midkine levels and (B) serum levels of protein IGF2 on thyroid cancer and (C) heparin‐binding EGF‐like growth factor levels on malignant neoplasm of thyroid gland.

**Figure:**
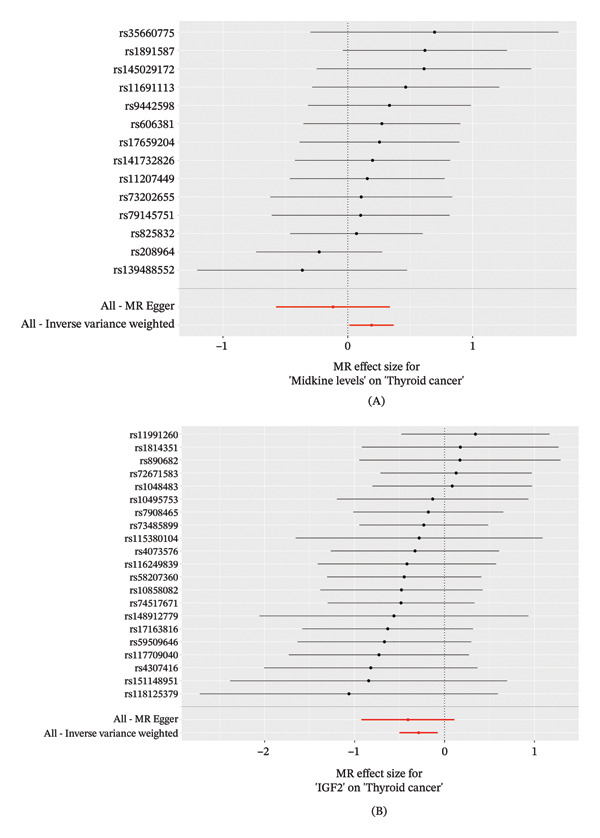

**Figure:**
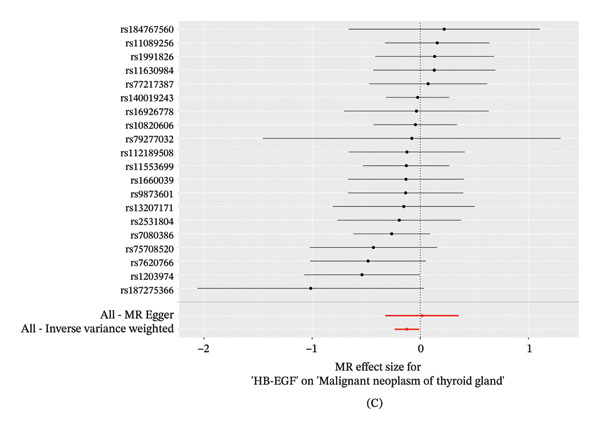

### 3.3. Sensitivity Analysis

Cochran’s Q statistics indicated significant heterogeneity in the association between FGF1 and malignant thyroid neoplasms (*Q* = 20.725, *p* = 0.036) (Table 2). Based on the comprehensive analysis results of this study, we deemed this bias tolerable. This is because the IVW method leverages the effects of multiple genetic variants on the target variable, which can mitigate biases potentially induced by genetic variation. MR‐Egger analysis found no evidence of horizontal pleiotropy in the association between heparin‐binding GDFs and thyroid cancer (all *p* > 0.05) (Table 2). Similarly, the MR‐PRESSO global test confirmed the absence of horizontal pleiotropy in these associations (all global *p* > 0.05) (Table 3). Leave‐one‐out analysis showed that no single SNP significantly influenced the causal relationship between heparin‐binding GDFs and thyroid cancer (Figures 4). Additionally, the symmetry of the funnel plots suggested no substantial heterogeneity in these associations (Figures 5).

**TABLE 2:** Assessing the heterogeneity and horizontal pleiotropy of heparin‐binding GDFs with thyroid cancer.

Exposure	Outcome	Heterogeneity		Horizontal pleiotropy	
		Cochran’s Q	*p*	MR‐Egger intercept	*p*
Thyroid cancer	Proneuregulin‐2	14.743	0.614	0.007	0.884
Thyroid cancer	Thyroglobulin levels	7.782	0.802	−0.017	0.613
Thyroid cancer	Insulin‐like growth factor 1 levels	16.590	0.551	0.032	0.393
Thyroid cancer	Heparin‐binding EGF‐like growth factor levels	20.838	0.346	0.069	0.078
Thyroid cancer	Midkine levels	8.985	0.774	0.053	0.176
Thyroid cancer	Pleiotrophin levels	6.101	0.942	−0.015	0.760
Thyroid cancer	Serum levels of protein IGF2	10.081	0.967	0.015	0.627
Thyroid cancer	Neuregulin‐1 levels	11.289	0.587	0.015	0.745
Thyroid cancer	Fibroblast growth factor 1 levels	18.162	0.111	0.080	0.088
Thyroid cancer	Serum levels of protein FGF2	7.608	0.667	0.073	0.160
Malignant neoplasm of thyroid gland	Proneuregulin‐2	9.357	0.858	0.012	0.772
Malignant neoplasm of thyroid gland	Thyroglobulin levels	9.932	0.536	−0.024	0.483
Malignant neoplasm of thyroid gland	Insulin‐like growth factor 1 levels	12.831	0.747	−0.009	0.781
Malignant neoplasm of thyroid gland	Heparin‐binding EGF‐like growth factor levels	13.215	0.827	−0.028	0.401
Malignant neoplasm of thyroid gland	Midkine levels	10.744	0.632	−0.016	0.589
Malignant neoplasm of thyroid gland	Pleiotrophin levels	17.240	0.189	0.033	0.514
Malignant neoplasm of thyroid gland	Serum levels of protein IGF2	18.242	0.506	0.001	0.970
Malignant neoplasm of thyroid gland	Neuregulin‐1 levels	7.207	0.891	−0.021	0.589
Malignant neoplasm of thyroid gland	Fibroblast growth factor 1 levels	20.725	0.036	−0.048	0.240
Malignant neoplasm of thyroid gland	Serum levels of protein FGF2	8.542	0.481	−0.027	0.566
Benign neoplasm of thyroid gland	Proneuregulin‐2	17.727	0.277	0.061	0.329
Benign neoplasm of thyroid gland	Thyroglobulin levels	13.864	0.241	−0.086	0.099
Benign neoplasm of thyroid gland	Insulin‐like growth factor 1 levels	22.140	0.179	0.030	0.565
Benign neoplasm of thyroid gland	Heparin‐binding EGF‐like growth factor levels	17.792	0.536	0.011	0.821
Benign neoplasm of thyroid gland	Midkine levels	5.915	0.949	0.055	0.212
Benign neoplasm of thyroid gland	Pleiotrophin levels	12.698	0.471	−0.100	0.118
Benign neoplasm of thyroid gland	Serum levels of protein IGF2	29.484	0.059	0.044	0.323
Benign neoplasm of thyroid gland	Neuregulin‐1 levels	17.316	0.185	−0.011	0.870
Benign neoplasm of thyroid gland	Fibroblast growth factor 1 levels	5.148	0.924	−0.016	0.697
Benign neoplasm of thyroid gland	Serum levels of protein FGF2	8.854	0.451	−0.061	0.360

*Note:* Cochran’s Q statistic is used for detecting heterogeneity about the IVW estimate.

**TABLE 3:** Detection and correction of horizontal pleiotropy using the MR‐PRESSO method.

Exposure	Outcome	Raw		Outlier corrected		Global P	Number of outliers	Distortion P
		OR (CI%)	*p*	OR (CI%)	*p*			
Thyroid cancer	Fibroblast growth factor 1 levels	1.0445 (0.8287–1.3166)	0.719	—	—	0.118	—	—
Thyroid cancer	Fibroblast growth factor 2 levels	0.9127 (0.7236–1.1513)	0.459	—	—	0.654	—	—
Thyroid cancer	Heparin‐binding EGF‐like growth factor levels	1.0698 (0.9272–1.2343)	0.367	—	—	0.280	—	—
Thyroid cancer	Insulin‐like growth factor 1 levels	0.9065 (0.7985–1.0292)	0.146	—	—	0.646	—	—
Thyroid cancer	Serum levels of protein IGF2	0.7334 (0.6300–0.8538)	0.179	—	—	0.968	—	—
Thyroid cancer	Midkine levels	1.2099 (1.0438–1.4024)	0.025	—	—	0.789	—	—
Thyroid cancer	Neuregulin‐1 levels	1.0111 (0.8660–1.1806)	0.891	—	—	0.651	—	—
Thyroid cancer	Proneuregulin‐2	0.9447 (0.8181–1.0908)	0.449	—	—	0.651	—	—
Thyroid cancer	Pleiotrophin levels	0.9104 (0.8159–1.0158)	0.117	—	—	0.936	—	—
Thyroid cancer	Thyroglobulin levels	1.0261 (0.9093–1.1579)	0.684	—	—	0.831	—	—
Malignant neoplasm of thyroid gland	Fibroblast growth factor 1 levels	1.0599 (0.8713–1.2894)	0.572	—	—	0.051	—	—
Malignant neoplasm of thyroid gland	Fibroblast growth factor 2 levels	1.1476 (0.9049–1.4555)	0.285	—	—	0.508	—	—
Malignant neoplasm of thyroid gland	Heparin‐binding EGF‐like growth factor levels	0.9051 (0.8132–1.0075)	0.083	—	—	0.617	—	—
Malignant neoplasm of thyroid gland	Insulin‐like growth factor 1 levels	1.0068 (0.9083–1.1159)	0.899	—	—	0.606	—	—
Malignant neoplasm of thyroid gland	Serum levels of protein IGF2	0.9574 (0.8034–1.1409)	0.632	—	—	0.483	—	—
Malignant neoplasm of thyroid gland	Midkine levels	1.0697 (0.9358–1.2228)	0.342	—	—	0.614	—	—
Malignant neoplasm of thyroid gland	Neuregulin‐1 levels	0.9351 (0.8380–1.0434)	0.252	—	—	0.916	—	—
Malignant neoplasm of thyroid gland	Proneuregulin‐2	0.9715 (0.8758–1.0777)	0.593	—	—	0.851	—	—
Malignant neoplasm of thyroid gland	Pleiotrophin levels	0.9493 (0.8097–1.1131)	0.533	—	—	0.224	—	—
Malignant neoplasm of thyroid gland	Thyroglobulin levels	0.8935 (0.7727–1.0332)	0.157	—	—	0.575	—	—
Benign neoplasm of thyroid gland	Fibroblast growth factor 1 levels	0.9266 (0.8076–1.0630)	0.300	—	—	0.939	—	—
Benign neoplasm of thyroid gland	Fibroblast growth factor 2 levels	1.1046 (0.7854–1.5534)	0.582	—	—	0.445	—	—
Benign neoplasm of thyroid gland	Heparin‐binding EGF‐like growth factor levels	0.9635 (0.8307–1.1174)	0.628	—	—	0.607	—	—
Benign neoplasm of thyroid gland	Insulin‐like growth factor 1 levels	0.9913 (0.8166–1.2035)	0.931	—	—	0.080	—	—
Benign neoplasm of thyroid gland	Serum levels of protein IGF2	1.1616 (0.8573–1.5739)	0.345	—	—	0.102	—	—
Benign neoplasm of thyroid gland	Midkine levels	0.9459 (0.8227–1.0875)	0.448	—	—	0.952	—	—
Benign neoplasm of thyroid gland	Neuregulin‐1 levels	1.1024 (0.8679–1.4004)	0.439	—	—	0.200	—	—
Benign neoplasm of thyroid gland	Proneuregulin‐2	1.0470 (0.8565–1.2798)	0.660	—	—	0.252	—	—
Benign neoplasm of thyroid gland	Pleiotrophin levels	0.9745 (0.8045–1.1804)	0.796	—	—	0.393	—	—
Benign neoplasm of thyroid gland	Thyroglobulin levels	0.9416 (0.7393–1.1991)	0.635	—	—	0.251	—	—

FIGURE 4The leave‐one‐out plot of the causal relationships of heparin‐binding GDFs with thyroid cancer. Each panel represents the causal estimates for (A) midkine levels and (B) serum levels of protein IGF2 on thyroid cancer and (C) heparin‐binding EGF‐like growth factor levels on malignant neoplasm of thyroid gland.

**Figure:**
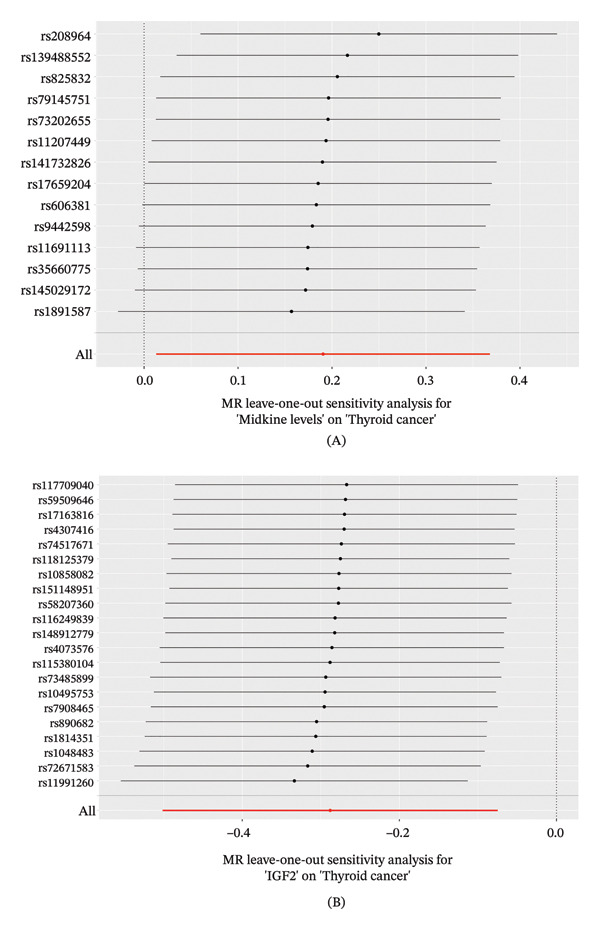

**Figure:**
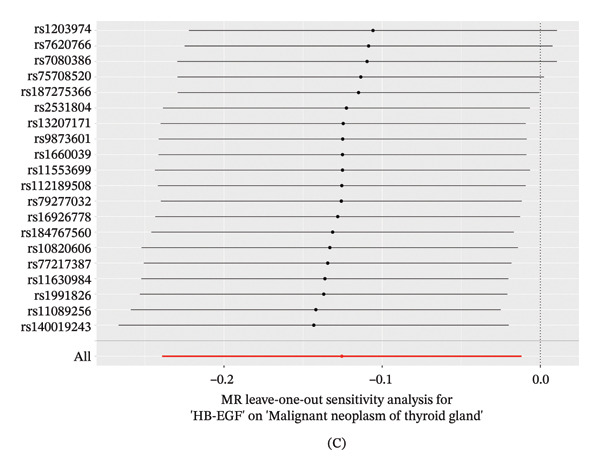

FIGURE 5Funnel plot of the causal relationships of heparin‐binding GDFs with thyroid cancer. Each panel represents the causal estimates for (A) midkine levels and (B) serum levels of protein IGF2 on thyroid cancer and (C) heparin‐binding EGF‐like growth factor levels on malignant neoplasm of thyroid gland.

**Figure:**
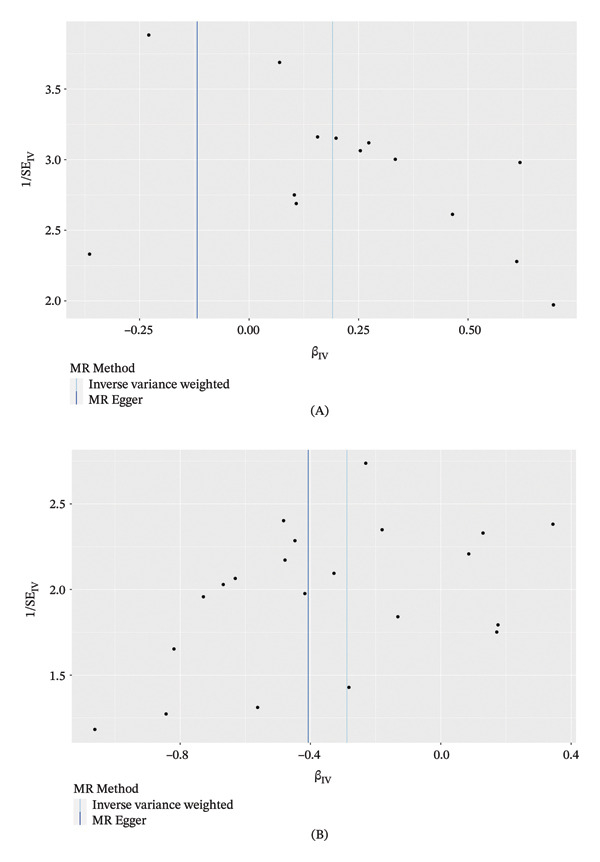

**Figure:**
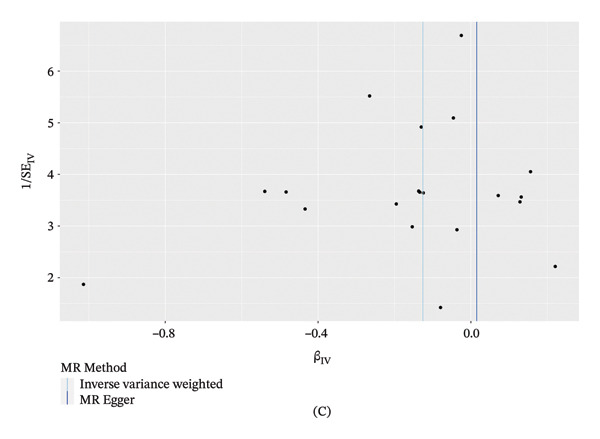

### 3.4. Differential Expression Analysis in Thyroid Tumor Tissues

**FIGURE 6:**
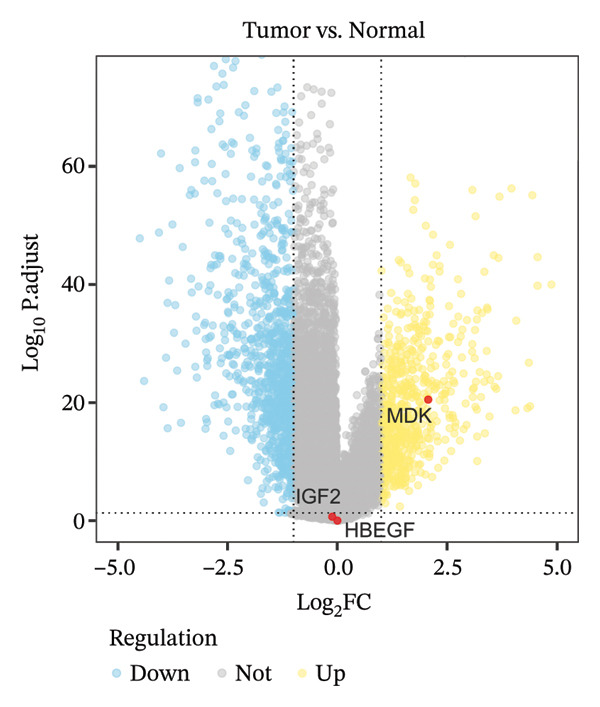
Volcano plot of differentially expressed genes in the TCGA–THCA cohort. The plot illustrates the relationship between the log2 fold change (logFC) and the statistical significance (‐log10 adjusted *p* value) for each gene. Red dots represent significantly upregulated genes (logFC > 0, p.adj < 0.05), blue dots represent significantly downregulated genes, and gray dots represent genes with no significant change. The gene MDK is highlighted, showing significant upregulation in tumor tissues.

To explore the expression patterns of the identified genes at the tissue level, we analyzed the TCGA–THCA cohort. The analysis revealed that *MDK* (the gene encoding midkine) was significantly upregulated in thyroid tumor tissues compared to adjacent normal tissues (logFC > 0, p.adj < 0.05). However, no significant differential expression was observed for IGF2 or HBEGF in tumor tissues (Figure 6).

## 4. Discussion

The IVW analysis revealed significant causal associations of midkine levels and IGF2 with thyroid cancer and between heparin‐binding EGF‐like growth factor and malignant thyroid neoplasms. No horizontal pleiotropy, outlier SNPs, and evident heterogeneity were reported, substantiating the robustness of our findings. Further research is needed to validate these results and explore the underlying mechanisms of thyroid cancer. It is worth noting that while the primary IVW method yielded statistically significant causal estimates, the alternative MR estimators (MR‐Egger, weighted median, and weighted mode) did not reach formal statistical significance (*p* ≥ 0.05). This discrepancy is a recognized phenomenon in MR studies and can be primarily attributed to differences in statistical power. The IVW method is the most statistically efficient estimator when horizontal pleiotropy is absent. Conversely, robust methods like MR‐Egger and weighted median relax the assumption of valid instrumentation to guard against pleiotropy, but they do so at the cost of statistical power, which typically leads to wider confidence intervals and larger *p* values, especially in analyses with a modest number of IVs. Importantly, our study demonstrated a clear directional consistency and showed no evidence of horizontal pleiotropy (as indicated by the nonsignificant MR‐Egger intercepts and MR‐PRESSO global test results). Under these conditions, the IVW estimates serve as the most reliable indicators of the potential causal effects, while the directional alignment of the alternative methods supports the qualitative stability of our findings.

The study revealed positive causal associations between midkine levels and thyroid cancer, suggesting that midkine may contribute to thyroid carcinogenesis. Midkine, known for its role in promoting cell proliferation, migration, and angiogenesis, has been implicated in various cancers [27, 28]. Its overexpression in thyroid cancer can be driving tumorigenic processes through similar mechanisms observed in other malignancies, such as enhancing cellular invasion and resistance to apoptosis [29]. Consistent with our findings, an Egyptian study revealed serum midkine levels significantly elevated as the thyroid tumor stage progressed [30]. To further investigate this connection at the tumor source, we examined its expression within the tissue itself. Our analysis of the TCGA–THCA dataset strongly supported this hypothesis, revealing that the mRNA expression of *MDK* is significantly upregulated in thyroid tumor tissues compared to adjacent normal tissues. This convergence of evidence—from a genetically predicted causal role for circulating levels to confirmed high expression at the tumor site—provides a compelling argument for the protumorigenic function of midkine in thyroid cancer, reinforcing its potential as a biomarker for further investigation.

However, the inverse association between IGF2 levels and thyroid cancer risk contrasted with the typically oncogenic role of IGF. IGF2 is a key regulator of growth and development, and its dysregulation has been linked to various cancers, including colorectal and breast cancer [31, 32]. A systematic review of the IGF bioregulation system in thyroid nodular disease found that IGF1 expression was consistently elevated in thyroid cancer, whereas IGF2 expression was generally lower in malignant tissue compared to normal thyroid tissue [33]. To explore whether this protective function is reflected at the tumor site, we analyzed its tissue‐level expression. Interestingly, our transcriptomic analysis of the TCGA–THCA cohort did not show significant differential expression of IGF2 within established tumor tissues. This apparent divergence between the effect of genetically predicted systemic levels and local tumor expression warrants careful interpretation. It suggests the protective effect of higher circulating IGF2 may occur during the early stages of carcinogenesis, rather than influencing the gene expression profile of a fully developed tumor. Alternatively, the causal effect could be mediated through systemic pathways that do not necessitate a change in IGF2 expression within the thyroid tumor itself [34–36]. This result challenges the conventional understanding of IGF2’s role in cancer and highlights the need for further investigation into its highly tissue‐specific functions in thyroid carcinogenesis.

A similar layer of complexity was observed for heparin‐binding EGF‐like growth factor, suggesting a protective role for this factor in malignant thyroid neoplasms. Heparin‐binding EGF‐like growth factor is involved in activating the EGFR signaling pathway, which is crucial for cell survival, proliferation, and differentiation [37]. Our findings contrasted with studies linking high levels of EGF‐like growth factors to poor prognosis in cancers such as glioblastoma and lung adenocarcinoma [38, 39]. Consistent with our observations for IGF2, our analysis of the TCGA dataset also found no significant changes in HBEGF expression in malignant thyroid neoplasms. This recurring pattern suggests that the protective causal effects inferred from our MR analysis for both these factors might not be directly linked to their transcriptional regulation within the established tumor microenvironment. Instead, their protective mechanisms could be systemic or primarily related to preventing the initial malignant transformation, processes not fully captured by a static gene expression snapshot of an established cancer. Further research is needed to elucidate the mechanisms underlying this protective association.

This study has several important limitations. The primary limitation is the reliance on GWAS data from populations of European descent. This constrains the external validity of our findings, a crucial point of caution given the well‐documented ethnic disparities in thyroid cancer incidence, particularly its higher prevalence in Asian populations. The genetic architecture of complex diseases can vary significantly across ancestries, including differences in allele frequencies, effect sizes, and LD patterns. Consequently, the causal estimates for midkine, IGF2, and HB‐EGF may not be generalizable to other ethnic groups. It is imperative that our findings be replicated in large‐scale MR studies conducted in diverse populations, especially in Asian cohorts, to ascertain whether these causal pathways are universal or population specific. Furthermore, our study utilized summary‐level data, which precluded subgroup analyses by age, sex, or specific cancer histotypes that might have uncovered heterogeneous effects. Finally, to ensure an adequate number of IVs for sensitivity analyses (such as MR‐Egger and MR‐PRESSO), we employed a relaxed *p* value threshold of < 5 × 10 ^−6^ for instrument selection. While this threshold is less stringent than the conventional genome‐wide significance level (*p* < 5 × 10^−8^) and could theoretically introduce weaker genetic instruments, we mitigated this risk by ensuring all included SNPs possessed an F‐statistic substantially greater than 10 (mean F‐statistic = 24.2). The lack of statistical significance in robust estimators like MR‐Egger might be partially influenced by the noise introduced under this relaxed threshold; however, the consistent directional estimates across all models and the absence of detectable pleiotropy suggest that the primary causal findings remain robust. Nonetheless, future studies with larger sample sizes and stronger genetic instruments meeting genome‐wide significance are warranted to confirm these relationships.

## 5. Conclusions

In summary, this MR study provides evidence for causal associations of genetically predicted midkine levels and IGF2 with thyroid cancer and of heparin‐binding EGF‐like growth factor with malignant thyroid neoplasms. These findings contribute to the understanding of thyroid cancer etiology and highlight directions for future mechanistic investigation.

**Nomenclature**
GDFs: Growth and differentiation factors
MR: Mendelian randomization
GWAS: Genome‐wide association studies
IVW: Inverse‐variance weighted
FGF1: Fibroblast growth factor 1
IGF1: Insulin‐like growth factor I
IVs: Instrumental variables
SNP: Single nucleotide polymorphism
MAF: Minor allele frequency
LD: Linkage disequilibrium

## Author Contributions

Conception and design: Xiwei Zhang and Ping Shi. Administrative support: Ping Shi and Yanzhao Wu. Provision of study materials or patients: Xiwei Zhang and Dongqiang Yang. Collection and assembly of data: Xiwei Zhang and Dongqiang Yang. Data analysis and interpretation: Xiwei Zhang and Ping Shi. Manuscript writing: Xiwei Zhang, Dongqiang Yang, Yan Liu, Yanzhao Wu, and Ping Shi.

## Funding

This study was supported by the Medical Science Research Project of Hebei (no. 20240378) and Hebei Provincial Department of Finance Government funded outstanding Medical talents project (ZF2025197).

## Disclosure

All authors gave final approval for the manuscript.

## Ethics Statement

The authors have nothing to report.

## Consent

The authors have nothing to report.

## Conflicts of Interest

The authors declare no conflicts of interest.

## Supporting Information

Additional supporting information can be found online in the Supporting Information section.

## Supporting information


**Supporting Information** Table S1. Overview of the data source. Table S2. Detailed information of IVs in the MR analysis of heparin‐binding growth/differentiation factors on thyroid cancer. Table S3. MR estimates of assessing the causal effects of heparin‐binding growth/differentiation factors on thyroid cancer.

## Data Availability

All data generated or analyzed during this study are included within this published article and its Supporting Information files.
