# Supplementary material for: Causal Associations of Heparin‐Binding Growth and Differentiation Factors With Thyroid Cancer: A Two‐Sample Mendelian Randomization Study
Source: Int J Endocrinol. 2026 Jul 15;2026:6165618. doi: 10.1155/ije/6165618 (PMC13373531; doi:10.1155/ije/6165618)
Supplement: Supplementary file 1 — Supporting Information Table S1. Overview of the data source. Table S2. Detailed information of IVs in the MR analysis of heparin‐binding growth/differentiation factors on thyroid cancer. Table S3. MR estimates of assessing the causal effects of heparin‐binding growth/differentiation factors on thyroid cancer. [file IJE-2026-6165618-s001.zip › Supplementary tables.docx]

Table S1. Overview of the data source.

| **Trait** | **GWAS ID** | **Sample size（case/control）** | **Number of SNPs** |
| --- | --- | --- | --- |
| Thyroid cancer | ebi-a-GCST90018929 | 1054/490920 | 24198226 |
| Malignant neoplasm of thyroid gland (all cancers excluded) | finn-b-C3_THYROID_GLAND_EXALLC | 989/174006 | 16380316 |
| Benign neoplasm of thyroid gland | finn-b-CD2_BENIGN_THYROID | 455/218337 | 16380466 |
| Fibroblast growth factor 1 levels | GCST90241161 | 3301 |  |
| Serum levels of protein FGF2 | GCST90088187 | 5368 |  |
| Insulin-like growth factor I levels | GCST90241514 | 3301 |  |
| Serum levels of protein IGF2 | GCST90089599 | 5358 |  |
| Neuregulin-1 levels | GCST90242060 | 3301 |  |
| Pro-neuregulin-2 | GCST90242352 | 3301 |  |
| Midkine levels | GCST90241936 | 3301 |  |
| Thyroglobulin levels | GCST90243017 | 3301 |  |
| Pleiotrophin levels | GCST90242297 | 3,301 |  |
| Heparin-binding EGF-like growth factor levels | GCST90241392 | 3,301 |  |

Table S3. MR estimates of assessing the causal effects of heparin-binding growth/differentiation factors on thyroid cancer.

| **Outcome** | **Exposure** | **Number of SNPs** | **Methods** | **OR (95% CI)** | **P** |
| --- | --- | --- | --- | --- | --- |
| Thyroid cancer | Pro-neuregulin-2 | 18 | MR Egger | 0.9146 (0.5807-1.4405) | 0.7052 |
| Thyroid cancer | Pro-neuregulin-2 | 18 | Weighted median | 0.9756 (0.7949-1.1974) | 0.8131 |
| Thyroid cancer | Pro-neuregulin-2 | 18 | Weighted mode | 0.9621 (0.677-1.3673) | 0.8321 |
| Thyroid cancer | Thyroglobulin levels | 13 | MR Egger | 1.0799 (0.8462-1.378) | 0.5493 |
| Thyroid cancer | Thyroglobulin levels | 13 | Weighted median | 1.0357 (0.8407-1.2759) | 0.742 |
| Thyroid cancer | Thyroglobulin levels | 13 | Weighted mode | 1.0336 (0.8361-1.2779) | 0.7652 |
| Thyroid cancer | Insulin-like growth factor I levels | 19 | MR Egger | 0.7892 (0.5696-1.0934) | 0.1728 |
| Thyroid cancer | Insulin-like growth factor I levels | 19 | Weighted median | 0.8325 (0.6873-1.0083) | 0.0608 |
| Thyroid cancer | Insulin-like growth factor I levels | 19 | Weighted mode | 0.825 (0.6319-1.077) | 0.1742 |
| Thyroid cancer | Heparin-binding EGF-like growth factor levels | 20 | MR Egger | 0.7408 (0.5022-1.0929) | 0.1478 |
| Thyroid cancer | Heparin-binding EGF-like growth factor levels | 20 | Weighted median | 1.0353 (0.8559-1.2523) | 0.7209 |
| Thyroid cancer | Heparin-binding EGF-like growth factor levels | 20 | Weighted mode | 0.9892 (0.7426-1.3177) | 0.9416 |
| Thyroid cancer | Midkine levels | 14 | MR Egger | 0.8883 (0.5625-1.4025) | 0.6203 |
| Thyroid cancer | Midkine levels | 14 | Weighted median | 1.1975 (0.9362-1.5318) | 0.1513 |
| Thyroid cancer | Midkine levels | 14 | Weighted mode | 1.1897 (0.8242-1.7172) | 0.3706 |
| Thyroid cancer | Pleiotrophin levels | 14 | MR Egger | 0.9901 (0.5716-1.7149) | 0.9723 |
| Thyroid cancer | Pleiotrophin levels | 14 | Weighted median | 0.9488 (0.7655-1.1761) | 0.6316 |
| Thyroid cancer | Pleiotrophin levels | 14 | Weighted mode | 0.9724 (0.7175-1.3179) | 0.8595 |
| Thyroid cancer | Serum levels of protein IGF2 | 21 | MR Egger | 0.6658 (0.397-1.1167) | 0.1397 |
| Thyroid cancer | Serum levels of protein IGF2 | 21 | Weighted median | 0.7377 (0.561-0.9702) | 0.0295 |
| Thyroid cancer | Serum levels of protein IGF2 | 21 | Weighted mode | 0.67 (0.4028-1.1144) | 0.1385 |
| Thyroid cancer | Neuregulin-1 levels | 14 | MR Egger | 0.9573 (0.6287-1.4575) | 0.8422 |
| Thyroid cancer | Neuregulin-1 levels | 14 | Weighted median | 0.9884 (0.7835-1.2468) | 0.9213 |
| Thyroid cancer | Neuregulin-1 levels | 14 | Weighted mode | 0.9386 (0.6274-1.4044) | 0.7629 |
| Thyroid cancer | Fibroblast growth factor 1 levels | 13 | MR Egger | 0.7389 (0.4859-1.1236) | 0.1848 |
| Thyroid cancer | Fibroblast growth factor 1 levels | 13 | Weighted median | 0.9521 (0.7241-1.2519) | 0.7253 |
| Thyroid cancer | Fibroblast growth factor 1 levels | 13 | Weighted mode | 0.9221 (0.5629-1.5104) | 0.7529 |
| Thyroid cancer | Serum levels of protein FGF2 | 11 | MR Egger | 0.5396 (0.2619-1.1118) | 0.1287 |
| Thyroid cancer | Serum levels of protein FGF2 | 11 | Weighted median | 0.8943 (0.627-1.2754) | 0.5374 |
| Thyroid cancer | Serum levels of protein FGF2 | 11 | Weighted mode | 0.6717 (0.3379-1.3353) | 0.2828 |
| Malignant neoplasm of thyroid gland | Pro-neuregulin-2 | 16 | MR Egger | 0.9229 (0.6406-1.3297) | 0.6733 |
| Malignant neoplasm of thyroid gland | Pro-neuregulin-2 | 16 | Weighted median | 0.9114 (0.7663-1.084) | 0.2945 |
| Malignant neoplasm of thyroid gland | Pro-neuregulin-2 | 16 | Weighted mode | 0.905 (0.6949-1.1787) | 0.4705 |
| Malignant neoplasm of thyroid gland | Thyroglobulin levels | 12 | MR Egger | 0.9763 (0.7355-1.2958) | 0.8712 |
| Malignant neoplasm of thyroid gland | Thyroglobulin levels | 12 | Weighted median | 0.8526 (0.6924-1.05) | 0.1334 |
| Malignant neoplasm of thyroid gland | Thyroglobulin levels | 12 | Weighted mode | 0.7895 (0.5735-1.0869) | 0.1752 |
| Malignant neoplasm of thyroid gland | Insulin-like growth factor I levels | 18 | MR Egger | 1.0306 (0.7946-1.3367) | 0.8229 |
| Malignant neoplasm of thyroid gland | Insulin-like growth factor I levels | 18 | Weighted median | 1.0734 (0.9054-1.2725) | 0.4146 |
| Malignant neoplasm of thyroid gland | Insulin-like growth factor I levels | 18 | Weighted mode | 1.147 (0.9229-1.4257) | 0.2331 |
| Malignant neoplasm of thyroid gland | Heparin-binding EGF-like growth factor levels | 20 | MR Egger | 1.0152 (0.7228-1.426) | 0.9315 |
| Malignant neoplasm of thyroid gland | Heparin-binding EGF-like growth factor levels | 20 | Weighted median | 0.8901 (0.7593-1.0434) | 0.1509 |
| Malignant neoplasm of thyroid gland | Heparin-binding EGF-like growth factor levels | 20 | Weighted mode | 0.9191 (0.738-1.1447) | 0.4605 |
| Malignant neoplasm of thyroid gland | Midkine levels | 14 | MR Egger | 1.1787 (0.8117-1.7117) | 0.4047 |
| Malignant neoplasm of thyroid gland | Midkine levels | 14 | Weighted median | 1.0904 (0.8897-1.3364) | 0.4046 |
| Malignant neoplasm of thyroid gland | Midkine levels | 14 | Weighted mode | 1.0937 (0.8087-1.4793) | 0.5707 |
| Malignant neoplasm of thyroid gland | Pleiotrophin levels | 14 | MR Egger | 0.7917 (0.4551-1.3772) | 0.4244 |
| Malignant neoplasm of thyroid gland | Pleiotrophin levels | 14 | Weighted median | 0.9828 (0.8112-1.1908) | 0.8595 |
| Malignant neoplasm of thyroid gland | Pleiotrophin levels | 14 | Weighted mode | 0.9934 (0.7949-1.2417) | 0.9548 |
| Malignant neoplasm of thyroid gland | Serum levels of protein IGF2 | 20 | MR Egger | 0.9711 (0.6333-1.489) | 0.8944 |
| Malignant neoplasm of thyroid gland | Serum levels of protein IGF2 | 20 | Weighted median | 0.9135 (0.711-1.1738) | 0.4796 |
| Malignant neoplasm of thyroid gland | Serum levels of protein IGF2 | 20 | Weighted mode | 0.8335 (0.5354-1.2977) | 0.4301 |
| Malignant neoplasm of thyroid gland | Neuregulin-1 levels | 14 | MR Egger | 1.0237 (0.7199-1.4558) | 0.8984 |
| Malignant neoplasm of thyroid gland | Neuregulin-1 levels | 14 | Weighted median | 0.8882 (0.7291-1.0819) | 0.2389 |
| Malignant neoplasm of thyroid gland | Neuregulin-1 levels | 14 | Weighted mode | 0.8944 (0.6583-1.2152) | 0.488 |
| Malignant neoplasm of thyroid gland | Fibroblast growth factor 1 levels | 12 | MR Egger | 1.2778 (0.9006-1.8129) | 0.1997 |
| Malignant neoplasm of thyroid gland | Fibroblast growth factor 1 levels | 12 | Weighted median | 1.0488 (0.8544-1.2874) | 0.6486 |
| Malignant neoplasm of thyroid gland | Fibroblast growth factor 1 levels | 12 | Weighted mode | 1.0481 (0.8417-1.3052) | 0.6827 |
| Malignant neoplasm of thyroid gland | Serum levels of protein FGF2 | 10 | MR Egger | 1.3779 (0.7206-2.6346) | 0.3608 |
| Malignant neoplasm of thyroid gland | Serum levels of protein FGF2 | 10 | Weighted median | 1.038 (0.7387-1.4586) | 0.8299 |
| Malignant neoplasm of thyroid gland | Serum levels of protein FGF2 | 10 | Weighted mode | 0.939 (0.5513-1.5992) | 0.822 |
| Benign neoplasm of thyroid gland | Pro-neuregulin-2 | 16 | MR Egger | 0.7997 (0.4571-1.3993) | 0.4467 |
| Benign neoplasm of thyroid gland | Pro-neuregulin-2 | 16 | Weighted median | 1.1406 (0.8733-1.4898) | 0.3342 |
| Benign neoplasm of thyroid gland | Pro-neuregulin-2 | 16 | Weighted mode | 1.3016 (0.8205-2.0647) | 0.2805 |
| Benign neoplasm of thyroid gland | Thyroglobulin levels | 12 | MR Egger | 1.2958 (0.8616-1.9489) | 0.2417 |
| Benign neoplasm of thyroid gland | Thyroglobulin levels | 12 | Weighted median | 0.8116 (0.5946-1.1078) | 0.1885 |
| Benign neoplasm of thyroid gland | Thyroglobulin levels | 12 | Weighted mode | 0.7999 (0.4709-1.3587) | 0.4263 |
| Benign neoplasm of thyroid gland | Insulin-like growth factor I levels | 18 | MR Egger | 0.9615 (0.6285-1.4709) | 0.8587 |
| Benign neoplasm of thyroid gland | Insulin-like growth factor I levels | 18 | Weighted median | 1.0798 (0.8377-1.3918) | 0.5535 |
| Benign neoplasm of thyroid gland | Insulin-like growth factor I levels | 18 | Weighted mode | 1.0702 (0.8053-1.4223) | 0.6459 |
| Benign neoplasm of thyroid gland | Heparin-binding EGF-like growth factor levels | 20 | MR Egger | 0.9179 (0.5682-1.4826) | 0.7302 |
| Benign neoplasm of thyroid gland | Heparin-binding EGF-like growth factor levels | 20 | Weighted median | 1.0101 (0.8104-1.2589) | 0.929 |
| Benign neoplasm of thyroid gland | Heparin-binding EGF-like growth factor levels | 20 | Weighted mode | 1.0438 (0.7683-1.4182) | 0.7868 |
| Benign neoplasm of thyroid gland | Midkine levels | 14 | MR Egger | 0.6845 (0.4053-1.1559) | 0.1816 |
| Benign neoplasm of thyroid gland | Midkine levels | 14 | Weighted median | 0.9585 (0.733-1.2534) | 0.7568 |
| Benign neoplasm of thyroid gland | Midkine levels | 14 | Weighted mode | 0.7532 (0.4693-1.209) | 0.2615 |
| Benign neoplasm of thyroid gland | Pleiotrophin levels | 14 | MR Egger | 1.6768 (0.8666-3.2445) | 0.1507 |
| Benign neoplasm of thyroid gland | Pleiotrophin levels | 14 | Weighted median | 1.0087 (0.7426-1.3701) | 0.9559 |
| Benign neoplasm of thyroid gland | Pleiotrophin levels | 14 | Weighted mode | 1.34 (0.8512-2.1096) | 0.2284 |
| Benign neoplasm of thyroid gland | Serum levels of protein IGF2 | 20 | MR Egger | 0.8113 (0.3853-1.7082) | 0.5888 |
| Benign neoplasm of thyroid gland | Serum levels of protein IGF2 | 20 | Weighted median | 1.0553 (0.722-1.5425) | 0.7811 |
| Benign neoplasm of thyroid gland | Serum levels of protein IGF2 | 20 | Weighted mode | 0.9681 (0.5749-1.6303) | 0.9042 |
| Benign neoplasm of thyroid gland | Neuregulin-1 levels | 14 | MR Egger | 1.1544 (0.6378-2.0896) | 0.6438 |
| Benign neoplasm of thyroid gland | Neuregulin-1 levels | 14 | Weighted median | 1.0199 (0.7559-1.376) | 0.8976 |
| Benign neoplasm of thyroid gland | Neuregulin-1 levels | 14 | Weighted mode | 0.9039 (0.5279-1.5477) | 0.7186 |
| Benign neoplasm of thyroid gland | Fibroblast growth factor 1 levels | 12 | MR Egger | 0.9866 (0.6834-1.4243) | 0.9441 |
| Benign neoplasm of thyroid gland | Fibroblast growth factor 1 levels | 12 | Weighted median | 0.9163 (0.6981-1.2028) | 0.529 |
| Benign neoplasm of thyroid gland | Fibroblast growth factor 1 levels | 12 | Weighted mode | 0.9032 (0.6485-1.258) | 0.5592 |
| Benign neoplasm of thyroid gland | Serum levels of protein FGF2 | 10 | MR Egger | 1.6732 (0.6758-4.1424) | 0.2981 |
| Benign neoplasm of thyroid gland | Serum levels of protein FGF2 | 10 | Weighted median | 1.0525 (0.6573-1.6853) | 0.8313 |
| Benign neoplasm of thyroid gland | Serum levels of protein FGF2 | 10 | Weighted mode | 0.9173 (0.4282-1.9652) | 0.8292 |
